# Heterogeneous Effects of Income on Physical and Mental Health of the Elderly: A Regression Discontinuity Design Based on China’s New Rural Pension Scheme

**DOI:** 10.3390/ijerph22111709

**Published:** 2025-11-13

**Authors:** Tao Ju, Mengmeng Pan

**Affiliations:** 1 School of Business, Anhui University, Hefei 230601, China; jutao19@mails.jlu.edu.cn; 2 Economics School, Anhui University, Hefei 230601, China

**Keywords:** aging, New Rural Pension Scheme, physical health, mental health, Regression Discontinuity Design method

## Abstract

Aging has been a social phenomenon unprecedented in history, which poses greater challenges on ensuring the health of the growing old population. We aim to estimate the effects of pension income on the physical and mental health of the elderly and further explore the complementary effects of external community medical environments with external pension income. We develop a Regression Discontinuity Design using an exogenous shock to the income—China’s New Rural Pension Scheme (NRPS), the world’s largest existing pension scheme. We find that public pension policy provides financial support to the elderly but also increases the loss of their perceived controllability. Specifically, empirical results indicate that pension income plays a positive effect on physical health and a negative effect on mental health. The positive effect only exists when communities have better medical environments, while the negative relationship is not affected by the external medical environment. Our findings reveal that internal pension income and external medical environment are therefore complementary factors to achieve better physical health of the elderly, while passive dependence on pension income may reduce mental health by heightening older people’s negative perceptions of losing controllability of their lives. Money is not omnipotent in both the physical and mental health of the elderly.

## 1. Introduction

The health of old people is a vital area of focus for policymakers around the world given the brutal reality that an aging wave is beginning to sweep across the globe. By 2050, 16% people in the world will be over age 65, up from 9% in 2019. Regions where the proportion of the population aged 65 years or over is projected to double between 2019 and 2050 include Northern Africa and Western Asia, Central and Southern Asia, Eastern and South-Eastern Asia and Latin America and the Caribbean [1]. In the pandemic of COVID-19, older people with underlying health conditions such as respiratory, cardiovascular disease and other chronic disease had much higher rates of infection and mortality than the rest of the population [2]. Given the irreversibility of aging trends, a wide variety of public welfare policies, supposed to be the best medicine for increasing the income of the elderly and thus improving their health, have been adopted by governments around the world [3]. Better understanding of the income–health gradient may lead to more effective pension programs as well as retirement policies. However, the causal effect of income on health remains a subject of considerable debate and empirical evidence presents a mixed picture. Therefore, employing an exogenous policy change in China’s pension system, this paper provides new and more robust evidence on the relationship between income and health.

On a positive note, Ettner [4] applied an instrumental variable approach to data on United States adults and identified a substantial positive effect of income on both physical and mental health. Similarly, Smith [5], utilizing the Panel Study of Income Dynamics dataset, demonstrated that socioeconomic status exerts a positive influence on health outcomes throughout the life course. In addition, findings by Frijters et al. [6] from a panel study of East and West Germans following reunification indicate that while the effect of income changes on health is statistically significant, its economic magnitude is relatively small. This positive relationship is also observed in developing economies; research from China, for instance, confirms that rising pension income can lead to significant improvements in health [7]. On the negative side, under different research backgrounds, a growing body of the literature finds that costly public welfare policies fail to improve the health of the elderly and even have a significant negative impact [8,9]. For example, Snyder and Evan [10] show that the American elderly who received less income in social security benefits had significantly lower mortality than those with higher earnings. Some people argue that because the above literature focuses on developed countries, it is not surprising to obtain no income–health gradient conclusion, given well-operating public health insurance and social security systems in those countries [11]. However, several studies on developing countries also find little evidence that income has a significant effect on health [12,13]. By investigating China’s Minimum Livelihood Guarantee (Dibao) and using propensity score matching to control for selection bias, Gao and Zhai [14] found that Dibao recipients were less optimistic about their economic prospects and less happy than their comparable non-recipient counterparts. Similarly, Qi and Wu [8] find that China’s minimum living security system produced significant welfare stigmatizing effects, which reduces recipients’ self-satisfaction, decreases the levels of happiness and self-confidence and results in poorer interpersonal relationships and self-evaluations. Finally, other studies have found mixed results: using lottery prizes as an exogenous source of income variation, Lindahl [15] show that higher incomes lead to better health for the full sample of the lottery players aged 34–76, but the income effect is not significant for those aged 60 and above.

In this article, we propose four possible reasons for volatility about the income–health gradient. First, in the past literature on the income–health gradient, sources of income vary. The early literature focuses on the total income of an individual or family [4,5,9]. After that, some of the literature looks for exogenous income shocks, such as the German reunification [6], winning the Nobel Prize [16] and lottery winnings [17]. The most far-reaching are the various welfare programs implemented by governments. For example, “Social Security Benefits Notch” in the U.S. [10], “the Minimum Livelihood Guarantee” in China [14] and various social pension programs around the world [3]. Among these three types of income, welfare policies are extremely special, and its starting point is usually to care for the vulnerable groups. However, the overprotection of vulnerable groups often leads to negative psychological barriers such as isolation and stigma effect [8,10,14]. Therefore, different sources of income may generate different effects on health.

Second, although quasi-experimental studies—examining phenomena from financial crisis [18], job displacement [19], lottery wins [16] and inheritances [20]—have attempted to isolate the causal impact of income on health, their findings are potentially confounded by covariates unrelated to income. For example, the generalizability of lottery studies is compromised by the non-random risk preferences of lottery players. Furthermore, the critical assumption of exogeneity is frequently untenable; lottery success may not be random with respect to health, and the receipt of an inheritance is conflated with the psychological trauma of bereavement, representing a violation of exclusion restriction.

Third, most studies focused on one aspect, physical or mental health [6,10,21]. However, physical health and mental health are not completely identical [22], and there may be a heterogeneous effect of income on different kinds of health of the elderly. Specifically, poverty often prevents older people from accessing healthcare services, leading to poor physical health outcomes, particularly in developing countries. Increasing income, of course, will increase the chances of improving their physical health [5,6,9]. While increased income is not an absolute determinant of mental health. On the contrary, it often brings about all kinds of psychological problems. For example, using the quasi experiment of Social Security Notch, Snyder and Evans [10] find that the high-income elderly are under great psychological pressure, since these people are less likely to take part-time jobs and thus experience social isolation. And China’s minimum living security system (Dibao) can be stigmatizing, which results in low self-respect, emotional depression and negatively affect the psychological health and well-being of the recipients [8]. Similarly, Gao and Zhai [14] find that Dibao recipients are less optimistic about their economic prospect and thus less happy than their non-recipient peers. Overall, mental health is more complex and difficult to control than physical health, thus these two kinds of health may respond diversely to the income stimulus.

Finally, in the COVID-19 crisis, external medical institutions and medical equipment have become the most important but extremely scarce goods and materials around the world. Without the basic medical facilities, increased income looks like making bricks without straw [23]. However, the critical role of external medical environment has been neglected in the previous literature. Obviously, income can only improve the financial ability of the elderly, and the external medical environment really determines whether they will receive sufficient healthcare services. Half the world lacks access to essential health services, and even in rich countries, not all people have access to the comprehensive services they need [22]. (According to the WHO, a survey of 11 high-income countries found that up to 41 percent of the elderly reported problems coordinating care. This problem is undoubtedly more acute in developing countries. In high-income, upper-middle-income, lower-middle-income and low-income countries, 51%, 51%, 70.7% and 91.9% respondents, respectively, said they are not accessing healthcare services due to transportation problems caused by the lack of convenient medical services in their communities and technical problems in medical services [22].) Therefore, in the absence of favorable external health environment, higher incomes do not necessarily improve the health of older people. Based on the above analysis, we address the controversial income–health effect from the following several aspects. First, we distinguish physical health from mental health and examine the heterogeneous effects of income on two types of health. Second, we are concerned about the moderating effect of external community health environment on income–health gradient.

**Figure 1:**
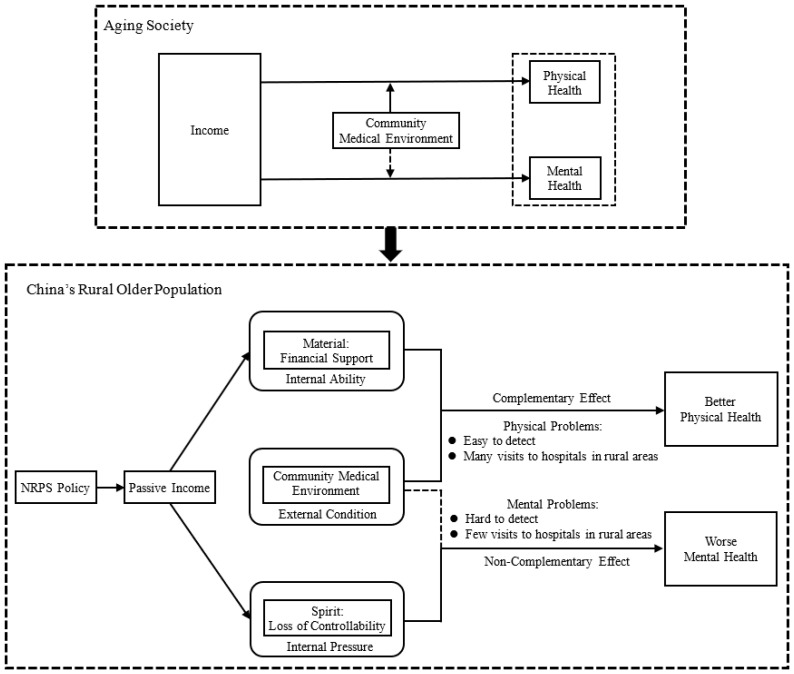
Conceptual framework.

This paper focuses on the Chinese market, a choice motivated by two key factors. Firstly, population aging is an irreversible global trend, and health issues among the elderly are ubiquitous, rendering research on the income–health nexus a matter of universal relevance. Furthermore, the challenges associated with population aging are more acute in developing nations. Specifically, by 2050, 80% of older people will live in what are now low- or middle-income countries, and nations like China and Brazil will have a greater proportion of older people than the USA [24]. And China, the largest developing country, is aging much faster than other low- and middle-income countries. Rural–urban migration contributes to rapid aging in rural areas: by 2030, the proportion of people aged 60 years or over in rural and urban areas will be 21.8% and 14.8%, respectively [25]. Second, China has the largest pension program in the world—China’s New Rural Pension Scheme (hereafter NRPS). Research on the health of China’s elderly population offers important lessons for other developing and developed nations, informing their respective policy and planning. Additionally, since the introduction of NRPS represents a quasi-natural experiment, the pension benefit is an exogenous shock to the income. We could therefore effectively address the endogeneity by applying a Regression Discontinuity design, which is the optimal method to analyze causality in the case of high time and economic costs of randomized trials. Overall, we focus on the following two questions. (1) Whether NRPS generates the heterogeneous effect on elderly’s physical and mental health? (2) Whether the impact of NRPS on health depends on convenient external medical environment? Based on the above analysis, we developed conceptual framework in Figure 1.

## 2. China’s New Rural Pension Scheme

China’s pension system has historically maintained a dual structure serving urban and rural residents separately. After the founding of the People’s Republic, China established its first formal pension system in 1951—an enterprise-based, pay-as-you-go scheme covering state employees. During this period, rural elders relied on the collective economic system and family support. Urban pension reforms commencing in 1978 gradually extended coverage to private sector workers by the 1990s, while rural economic transitions simultaneously eroded the collective support framework, leaving families as the primary source of old-age security [26]. The government initiated rural pension pilot programs in 1986, prioritizing individual contributions supplemented by collective funding, which resulted in low participation rates. Although these pilots expanded geographically during the 1990s, concerns regarding financial sustainability persisted, prompting the government to halt expansion in 1998. By 2000, census data indicated that fewer than 5% of rural elderly received pension benefits, while approximately 43% continued working and 48% depended on family support [27].

To rectify these disparities, the Chinese government launched the New Rural Pension Scheme (NRPS) in 2009. This nationwide initiative expanded rapidly from initial implementation in 320 pilot counties to near-universal coverage by 2012, reaching almost all of China’s 2853 counties. According to the China Statistical Yearbook 2012, the program enrolled 326.4 million rural participants, accounting for nearly one-quarter of the country’s population, with 89.2 million beneficiaries receiving pensions by 2011. The NRPS integrates a social pension component with a contributory scheme to provide basic income protection for the elderly while encouraging personal savings. Since our study focuses primarily on beneficiaries of the non-contributory element, subsequent sections will detail the social pension’s key features.

The scope of the NRPS is the rural residents who have reached the age of 16 and have not participated in the basic urban pension scheme. Individual contributions are voluntary, and the premiums are categorized into five tiers: CNY 100, 200, 300, 400, and 500 per year per person and the local governments are required to match CNY 30 annually per individual contribution. Participants who have contributed for at least 15 years will be qualified to receive pension benefits at age 60, which consists of a monthly payment from the individual account and a non-contributory basic pension. Rural residents aged 60 and over at the start of the program are eligible to directly receive the non-contributory basic pension (CNY 55), if their eligible adult children enroll in the scheme. The age-60 eligibility threshold provides the exogenous discontinuity necessary for a Regression Discontinuity (RD) design. It can be seen from the composition of pension treatment that the main source of the current new rural insurance pension is the basic pension. And the basic pension part is completely borne by the government financial burden, which means that the new rural insurance pension has a strong public transfer payment nature.

The distribution of pension benefits is administered by local governments, with practices varying significantly across regions. In more developed provinces such as Jiangsu and Zhejiang, pensions are typically transferred automatically into individual bank accounts established for each beneficiary. In contrast, in less developed areas, pensioners or their family members are often required to collect payments in person at designated village offices. To prevent corruption and ensure transparency, the NRPS operates under strict funding regulations. Local governments are obliged to submit annually updated personal information to the central government. In cases where pensioners are elderly, infirm, bedridden, or reside in remote locations, their children may collect the pension on their behalf, provided they can furnish proof that the beneficiary is still alive. Acceptable forms of verification include a recent video recording, or an official certificate issued by a local government representative who has conducted a recent in-person visit.

The existing literature has examined the impacts of NRPS from multiple angles. In terms of direct effects, studies have assessed both the absolute influence of the NRPS on health outcomes [28] and its role in reducing health inequality among the elderly [29]. In addition, Ning et al. [30] found no significant evidence that NRPS benefits induce older adults to exit the labor market; Cheng et al. [31] reported a positive effect of pension income on independent living among beneficiaries. Beyond direct outcomes, a growing body of research explores the spillover effects of the NRPS. Zheng et al. [32] demonstrated its intergenerational impact, showing a significant association between pension coverage and improved health status among children under 15. Similarly, by altering lifetime budget constraints, the NRPS has been found to influence fertility decisions: Shen et al. [33] documented a significant reduction in the number of children and a lower likelihood of having a second child following the scheme’s expansion. Further spillover effects extend to household energy choices. Ren and Xiong [34] showed that the NRPS encourages a shift from traditional biomass fuels, such as firewood, toward cleaner energy sources like electricity. Together, these studies illustrate the broad and multifaceted influence of pension policies on economic behavior, health, and environmental outcomes in rural China.

## 3. Materials and Methods

### 3.1. Materials

The data in this study are from the China Health and Retirement Longitudinal Study (CHARLS), which is a biennial survey in China being conducted by the National School of Development (China Center for Economic Research) at Peking University. CHARLS aims to be representative of the residents of China age 45 and older, with no upper age limit. The national baseline sample size is 10,287 households and 17,708 individuals, covering 150 counties in 28 provinces. The baseline of the CHARLS pilot took place in two provinces in the fall of 2008 and the data are public on the CHARLS website. The first national baseline wave was fielded from late summer 2011–March 2012; Wave 2 was in 2013 and Wave 3 was in 2015. Considering that in 2012, the new rural insurance achieved nationwide coverage, in this paper, we use the Wave 2 in 2013 and Wave 3 in 2015, which ensures that there will be no regional selection bias in sample selection. Specifically, we take the survey data of 2015, which included the largest number of respondents, as the benchmark, and included the respondents that existed in 2013 and died in 2015. The national survey in 2015 included over 20,967 elders, 12,144 families and 450 communities, and the number of respondents existed in 2013 and died in 2015 is 830. We excluded respondents who participated in other business insurance program other than the NRPS, because having other types of business insurance also affects the health of the elderly and the inclusion of these respondents is likely to skew the estimates. After deleting all the missing values of the variables involved, we finally obtained a sample of 4846 elderly people. The missing values of physical health and the variable of whether receiving pension income are the main reasons for the sharp drop from total sample to our final sample.

### 3.2. Variables and Measures

Physical health (PHealth). Self-comment health status is a valid indicator for measuring physical health [35]. In the CHARLS database, the indicators of self-assessment of physical health are divided into two criteria. The one is “Would you say your health is (1) very good, (2) good, (3) fair, (4) poor or (5) very poor”. Another one is “Would you say your health is (1) excellent, (2) very good, (3) good, (4) fair, (5) poor”. Half of the respondents followed the first criterion, and the other half followed the second. For uniform caliber, we used the first standard due to more even options. We used reversed coding, which is (1) very poor, (2) poor, (3) fair, (4) good or (5) very good, which means that the higher value represents better self-rated physical health.

Mental health (MHealth). The mental health score of this paper was calculated from the center for the epidemiological studies depression scale (CES-D10) [36]. The CES-D10 scale included 2 positive emotions, 5 somatic symptom items and 3 depressive mood items. Positive mood items like “I Felt Hopeful About the Future”. Somatic symptom items such as “I Felt Everything I Did Was an Effort”. Depressive mood items like “I Felt Lonely”. Respondents were asked to judge each item, choosing from “Few or None at All (<1 day)”, “Not Much (1 day to 2 days)”, “Sometimes or Half the Time (3 days to 4 days)” and “Most of the Time (5 days to 7 days)”. Referring to the existing literature, we assigned the above options for depression and somatic symptoms to integers of 4 to 1, respectively, and the above options for positive emotions to opposite values. The total score of the 10 items reflects the degree of mental health, with the score ranging from 10 to 40. The higher the score, the better the mental health.

Whether to receive pension income (NRPS) stipulates that rural residents aged 60 and over at the start of the program are eligible to directly receive the non-contributory basic pension. NRPS equals 1 means that the group is a treatment group, in which the older people receive pension income every month; otherwise, NRPS equals 0.

Community medical environment (MEnvironment). We use the number of community hospitals to measure medical environment. In the CHARLS database, it counts the number of different types of hospitals in each community. There are mainly the following categories: (1) General hospitals; (2) Specialized hospitals; (3) Chinese medicine hospitals; (4) Nearby pharmacy store; (5) Community healthcare center; (6) Community healthcare medical post; (7) Township health clinic hospital; (8) Village medical post. There is no doubt that the better the community’s medical environment is, the more convenient the elderly can access medical services, if there are more medical institutions in a community. We first calculate the total number of these hospitals, then group them by the median number of community hospitals. If the number of community hospitals is above the median, we consider the group with better medical environment, otherwise, with worse medical environment.

Referring to prior research, we have controlled the following variables that might affect the health of the elderly from the individual and community level. Specifically, as for the individual level, we control variables such as gender, marital status, the number of surviving children, other income except NRPS pension and the transfer payments received from children. For community-level variables, we control the quantity of tap water and the economic level [7].

### 3.3. Model

Based on the characteristics of the NRPS design, we use Regression Discontinuity Design (RDD) to examine the causal effect of the new rural insurance pension on elderly health. Among the quantitative methods used to identify causal effects, RDD has emerged and been widely used in the last decade. The basic idea of this identification strategy is to make use of the discontinuous characteristics on the policy rules such that the individual economy will be treated when a certain observable characteristic variable (running variable) is equal to or greater than a certain threshold. If the individual cannot completely control the running variable, the discontinuous change in the dependent variable can be considered as caused by the policy. According to the guideline of NRPS, pension benefits are paid according to the age rule, which states that only those who have reached the age of 60 can receive pension benefits:

(1) $$NRPS_{i}=\left\{\begin{array}{l} 1\;\;\;z_{i}\geq 60\\ 0\;\;\;z_{i}<60 \end{array}\right.$$

where *NRPS_i_* is the treatment status state variable, indicating whether a pension is received or not; *NRPS_i_* equals 1, indicating a pension is received; otherwise, indicating no pension. Equation (1) shows that *NRPS_i_* is a discontinuous function of age *z_i_*, and 60 years is the breakpoint, that is, no matter how close to 60 *z_i_* is, *NRPS_i_* will not change until *z_i_* is equal to 60. In RDD, the variable *z_i_* is called the running variable. If Equation (1) is true, the causal impact of pension income on health can be obtained by regression of the following Equation (2):

(2) $$Y_{i}=\alpha +\beta NRPS_{i}+f(z_{i})+\varepsilon_{i}$$

where *f*(*z_i_*) is a polynomial function. In many cases, the treatment status variable *NRPS_i_*, although a discontinuous function of the running variable *z_i_*, does not necessarily change from 0 to 1 at the cut point, but only increases the probability that *NRPS_i_* equals 1. So, *NRPS_i_* and *z_i_* have the following relationship in Equation (3):

(3) $$P\left[NRPS_{i}=1\;|\;z_{i}\right]=\left\{\begin{array}{l} g_{1}(z_{i})\;\;\;z_{i}\geq 60\\ g_{0}(z_{i})\;\;\;z_{i}<60 \end{array}\right.,\;g_{1}(z_{i})\neq g_{0}(z_{i})$$

In the background of this study, we assume *g*_1_(*z_i_*) > *g*_0_(*z_i_*), that is, people 60 and older are more likely to receive a pension than people younger than 60, which is a very reasonable assumption. Due to factors such as non-participation or interrupted contribution histories, some elderly individuals are ineligible for pension benefits even after turning 60. This situation renders the age-60 cutoff a fuzzy, rather than a sharp, regression discontinuity. Therefore, we use fuzzy RDD to estimate the causal effect. Since local governments tend to pay pensions centrally at the end of the year, the cut point is not exactly at the age of 60. In this study, we calculate the age accurately to the month and use 60.5 as the cut point in the regression analysis instead of 60 [37]. The optimal bandwidth is calculated according to Imbens and Kalyanraman (IK method) [38]. The essence of the IK method lies in its selection of the optimal bandwidth through the minimization of the Mean Squared Error (MSE). This minimization is achieved by balancing the trade-off between the two components of MSE: squared bias and variance. The squared bias is typically estimated using a quadratic term, while the variance estimate relies on the root mean square of the residuals. The estimation results of multiple bandwidth settings near the optimal bandwidth are reported to fully demonstrate the robustness of the results.

### 3.4. Summary Statistics

Table 1 reports the variables definitions and descriptive statistics. The sample consists of 4846 observations. Compared to those who have not received pension income, 2101 (43.4%) respondents begin to receive pension benefits. The mean of PHealth among individuals not receiving NRPS pension is 3.18, and this value in receiving NRPS pension group is 3.03. And the average of MHealth in no receiving NRPS pension group is 32.17, while this value in receiving NRPS pension group is 30.81. The above data show that those who receiving pension income have worse physical and mental health than that of not receiving pension income. This is reasonable. Because in terms of age, the average age of respondents in no receiving NRPS pension group is 52.28, which is much lower than that in the receiving NRPS pension group (67.53). Table 1 also provides statistics of variables at the individual level and community level.

## 4. Empirical Results

### 4.1. Graphical Analysis

**Figure 2:**
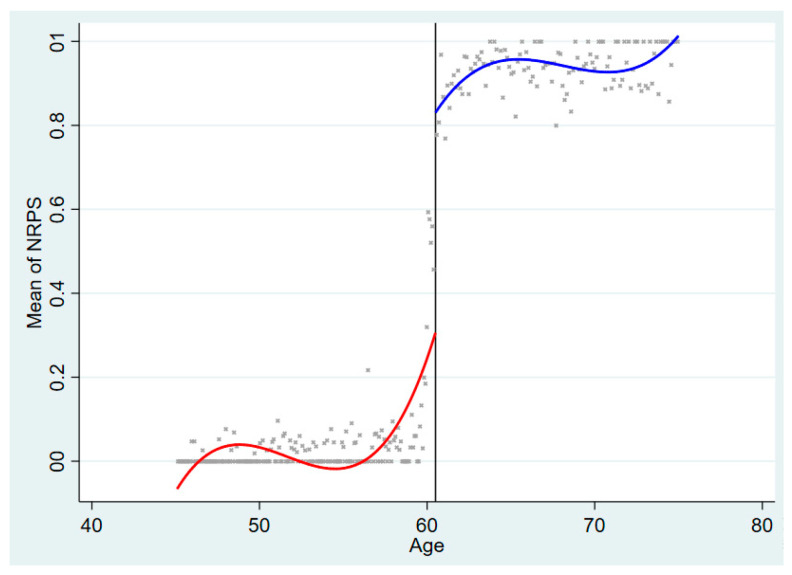
The probability that each age population receives pension benefits.

Before the regression analysis, we visually demonstrated the relationship between the running variable (Age) and the treatment status variable (NRPS) and the outcome variable (PHealth and MHealth) in the form of a graph. Figure 2 shows the relationship between the running variable Age and whether to receive pension benefits, which corresponds to the first stage in the Fuzzy RDD estimation. According to Figure 2, the pension receiving rate of the elderly has a significant upward jump at the cut point, which is consistent with the analysis above. Since the other characteristics of the elderly are similar near the cut point, the change in the outcome variables is completely caused by the policy, which effectively alleviates the endogeneity problem in the previous literature [16,20].

**Figure 3:**
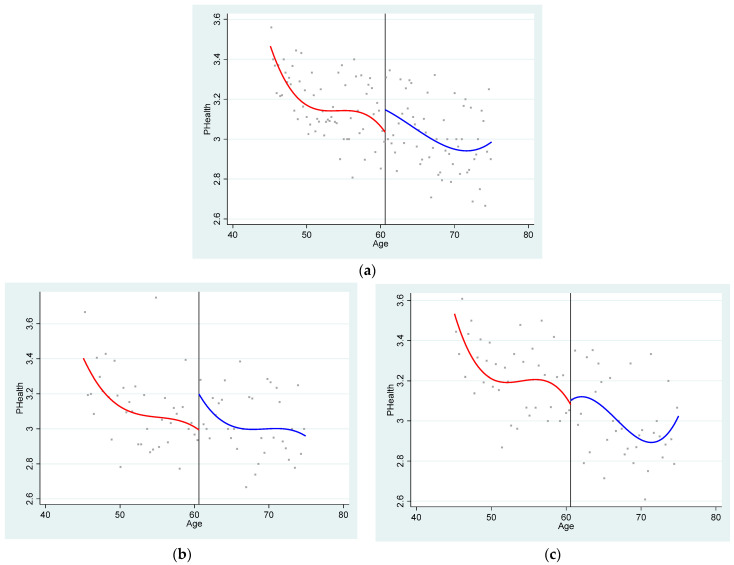
(**a**) The relationship between physical health status and age of population of all ages. (**b**) The relationship between physical health status and age in better community medical environment. (**c**) The relationship between physical health status and age in worse community medical environment.

Figure 3a shows the relationship between the running variable and physical health, which corresponds to the reduced result in the Fuzzy RDD estimation. Again, we observe a significant increase in the physical health of the elderly at the cut point. This preliminary result indicates that the new rural insurance pension income can improve the physical health of the elderly in rural areas. An increase in income can improve material living conditions and nutritional intake, enhance housing and physical environments and promote healthy behaviors and lifestyles—all of which contribute to better physical health among the elderly, which are consistent with previous research [7,9]. In Figure 3b,c, we further describe the relationship between pension income and the physical health of the elderly in different healthcare environment. We can find that the significant upward jump of physical health still exists when the community has better healthcare environment, while the upward jump of physical health nearly disappears when the community has worse healthcare environment. This means that pension income combined with community health facilities can have a positive impact on the physical health of the elderly. Unlike the previous literature that solely emphasizes the role of income [28,29], this study finds that for the elderly—a population with high morbidity—accessible external healthcare conditions are equally crucial [22,23]. Even with rising income levels, the lack of adequate medical infrastructure may still leave older adults struggling to access necessary healthcare services.

On the other hand, as can be seen from the scatter plot, the health status of the elderly declines slowly with age in communities with good medical conditions, while the health status of the elderly declines rapidly with age in communities with worse medical conditions, which also indicates the importance of medical service to the elderly. It is noteworthy that in regions with poorer medical conditions, the physical health of the elderly shows improvement around the age of 70. A possible explanation for this is that many elderly people in rural China may only be freed from heavy farm labor after their 60s, allowing them more time and energy to focus on their health. This positive change in lifestyle, with its cumulative effects becoming apparent after the age of 70, helps stabilize or even improve certain physiological indicators [39]. Due to limitations in medical resources, they can only achieve these effects through self-regulation. Additionally, the survivor selection effect may be another explanation. The least healthy portion of the peer group may have passed away before the age of 70, thus pulling up the average health level of the surviving population [40].

**Figure 4:**
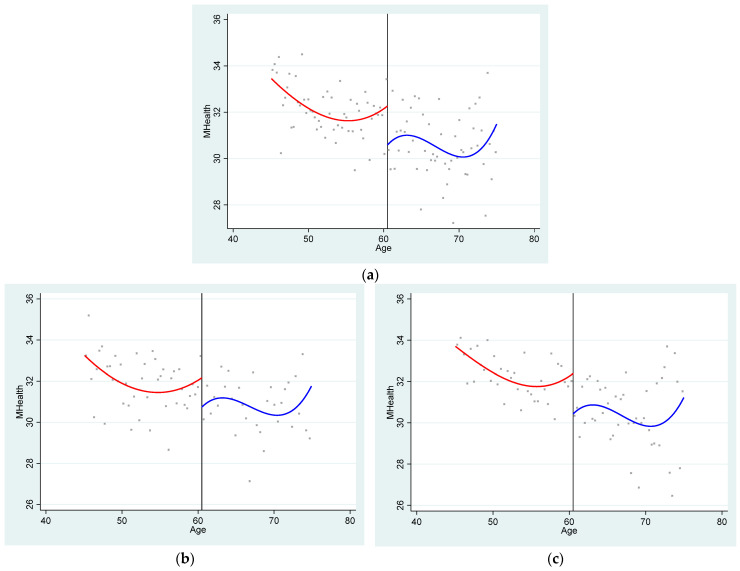
(**a**) The relationship between mental health status and age of population of all ages. (**b**) The relationship between mental health status and age in better community medical environment. (**c**) The relationship between mental health status and age in worse community medical environment.

Figure 4a shows the relationship between the running variable and mental health. Different from physical health, mental health level has a significant downward jump at the cut point, indicating that the new rural insurance pension income has a negative effect on the mental health of the elderly in Chinese rural areas. This finding contrasts with earlier studies [16,20]. On one hand, previous research has often focused on income from specific sources such as lottery winnings [17], Nobel Prize awards [16], or the Minimum Livelihood Guarantee [14]. Pension policies, however, represent a distinct category. They are fundamentally designed to protect vulnerable groups. Yet, excessive protection can inadvertently foster adverse psychological effects, including social isolation and stigma [8,10,14]. Consequently, the source of income may be a critical factor in determining its ultimate impact on health. On the other hand, this paper employs a regression discontinuity design, which enables a cleaner identification of causal effects and yields more robust empirical findings. Figure 4b,c describe the relationship between pension income and the mental health of the elderly in different healthcare environments. This downward jump exists in both groups. This finding is consistent with our expectation and the results presented in Figure 4. The explanation lies in the fact that pension income may intensify a negative psychological perception among the elderly: the belief that they “can no longer rely on their own labor for a living.” This cognitive bias, rooted in a lifelong engagement with physical work, forms an ingrained mentality among rural seniors that is difficult to alleviate through external means such as medication. The above results preliminarily verify the hypothesis of our paper, and we will verify it more accurately by means of regression analysis.

### 4.2. Regression Analysis

The graph above clearly shows the discontinuous changes in treatment status variable (NRPS) and outcome variables (PHealth and MHealth) caused by the policy regulation, as well as the impact of the community medical environment. However, whether these effects really exist and how big they are still depends on the results of parameter estimation [41].

First, Table 2 reports the RDD regression results of NRPS on physical and mental health of the elderly. In the first column of the table, the optimal bandwidth calculated according to Imbens & Kalyanaraman [38] is used for estimation. In the second to fourth columns, other similar bandwidth settings are used to fully test the stability of the results. The casual pathway from Age to NRPS is shown in Panel A. Age represents the impact of the new rural insurance policy’s age rule on whether to receive pension benefits or not. The coefficients of Age are positive and significant in all bandwidths. Therefore, we received the same results, and meeting the pension age requirement significantly increased the probability of receiving pension benefits. NRPS represents the results of the second stage, which is whether the new rural insurance pension income will improve the physical health of the elderly. Based on the bandwidth of +/− 2.5, +/− 2.0 and +/− 3.0, the coefficients of NRPS are positive and significant (1.500 with t-value = 1.930, 2.687 with t-value = 1.695 and 0.958 with t-value = 1.799, respectively). While at the bandwidth of +/− 4.0, the coefficients of NRPS are positive and marginally significant (0.396 with t-value = 1.183). Overall, pension income improved the physical health of the elderly under different bandwidths.

Panel B in Table 2 reports the effect of pension income on mental health of the elderly. Similarly, under all bandwidths, the probability of receiving pension benefits increases at the age cut point. For mental health, an opposite result to physical health can be seen and the coefficients are all negative under four bandwidths and significant at the bandwidth of +/− 2.8, +/− 3.0 and +/− 4.0, which indicate the mental health of the elderly experience a significant decrease at the cut point. The regression results are consistent with those shown in the graph above.

Second, to further investigate the joint effect of the medical environment and NRPS on health, we re-estimate the effect of pension income on health under different community medical environment. Table 3 reports the relationship between pension income and physical health under different medical environment. In Panel A, the coefficients of NRPS on PHealth are all positive and significant at 5% level under different bandwidths when the community has a better medical environment, while the coefficients of NRPS on PHealth are not significant in Panel B when the community has a worse medical environment. In addition, under the same bandwidths, the coefficients of NRPS are significantly larger in the group with better medical environment than those in the group with a worse medical environment. The results show that without the assistance of community healthcare, the pension itself could hardly have a positive impact on the physical health of the elderly.

Table 4 reports the relationship between pension income and mental health under different medical environment. In Panel A, the coefficients of NRPS on MHealth are −4.400, −7.007, and −4.976, respectively, and all significant at 10% level for those in community with better medical environment in +/− 4.4, +/− 3.0, and +/− 4.0 bandwidths. In Panel B, the coefficients of NRPS on MHealth are negative and significant under the bandwidths of +/− 3.5 and 4.0 and negative and marginal significantly under the bandwidth of 3.0 when the community has a worse medical environment. Since we did not receive good results in the first stage at bandwidth +/− 2.0, the regression results in the second stage are not significant. The results show that the negative relationship between income and mental health is not affected by the medical environment.

### 4.3. Validity Check

First, one way to test the validity of RDD estimation is to test the continuity of the predetermined control variables [38]. Intuitively, the treatment status variable (NRPS) should not influence what has happened before the age of 60.5. We test the continuity of concomitant variables in Table 5. The regression setting is the same as the previous regression model, except that the dependent variable is no longer the health level, but the original concomitant variables. The coefficients of all concomitant variables are not significant, which show that the policy has no significant effect on some characteristic variables of the elderly and the community.

Second, Placebo Test. Specifically, we limited the sample to the respondents in their communities who have not yet carried out the new rural insurance pilot, and used the same model setting for estimation to estimate the effect of age rules on the health of the elderly. For this group of respondents, no pension benefit is possible regardless of whether they reach the policy pension age, so we should not see any significant results. By 2012, all 2853 county-level administrative regions in China had launched trials of the new rural insurance system. Therefore, we selected the survey data of CHARLS database in 2011, when there are still some regions that have not carried out the pilot program of new rural insurance. Table 6 reports the results of the falsification test. The results show that the age rule has no significant effect under all model settings, which further illustrates the validity of RDD estimation.

## 5. Discussion

This is the first study to explore the diverse effects of pension income and its interaction with external medical environment on the physical and mental health of the rural elderly in China. Understanding the consequences of pension income is a critical issue for evaluating the effectiveness of public welfare policies, given the high burden of chronic disease in Chinese older population. It reports that chronic diseases now account for an estimated 80% of total deaths and 70% of total disability-adjusted life-years lost [42]. Using the two waves of CHARLS survey data and an RDD method, we study how NRPS affects the two kinds of health of the rural elderly. We find that the elderly who receive pension income have better physical health but worse mental health to their peers who receive no pension income. The positive effect of pension income, however, only exists when communities have better medical environments, while the negative relationship of pension income is not affected by external medical environments. Therefore, there are four aspects of our study that need to be discussed.

Firstly, the results in Panel A of Table 2 indicate that pension income improves the physical health of the elderly, a finding consistent with the prior literature [7,9]. The inability to afford hospitalization costs is a primary barrier to healthcare access for older populations, particularly in developing countries [22]. The Chinese context is illustrative: prior to 2009, a formal pension program was absent, and rural areas have long grappled with the dual challenges of high medical costs and limited-service availability. This situation is rooted in deeper social trends. For millennia, the traditional concept of “raising children for old age” prevailed in China [43], yet the recent emergence of a “NEET group” (Not in Education, Employment, or Training) has undermined the viability of relying on offspring for support [44]. Concurrently, the massification of higher education has increased financial pressures on new graduates, often leading parents to subsidize their adult children’s living expenses. These phenomena have collectively eroded the capacity of rural elderly to afford medical care. The implementation of the NRPS has thus provided essential basic living security and facilitated access to healthcare, demonstrating a positive welfare effect on the physical health of the elderly. Our results suggest that, against the backdrop of the NRPS’s nationwide rollout, the Chinese government should consider progressively increasing pension benefits for the elderly.

Secondly, the results in Panel B of Table 2 indicate a significant decline in mental health among the elderly following pension receipt—a counterintuitive yet explainable finding. Like the stigma effects observed with China’s Minimum Living Security System [8,14], the New Rural Pension Scheme (NRPS) may create psychological dilemmas that negatively impact mental well-being. Specifically, passive dependence on pension income appears to amplify negative self-perceptions regarding loss of control and autonomy. This phenomenon is particularly salient in rural China, where self-sufficiency has long been central to agricultural life and cultural identity. The traditional belief that “diligent hands shape one’s destiny” has sustained generations through agricultural labor, making the transition to passive pension receipt psychologically disruptive. The monthly payments fundamentally challenge deeply held values of self-reliance, contradicting the ethos that one should “depend on neither heaven nor earth, but only on one’s own hands”.

Self-determination theory helps explain this dynamic, emphasizing the importance of autonomous motivation and perceived control for psychological well-being [45]. Upon reaching pension eligibility at age 60, the shift to non-labor income may reinforce negative self-perceptions about aging and diminished autonomy. Substantial evidence links such loss of control to adverse mental health outcomes, including cardiometabolic risks, anxiety disorders, and depression [46,47,48]. The classic nursing home study by Langer and Rodin [49] powerfully demonstrates this principle: residents who maintained control over their daily lives showed significantly better survival rates—with mortality rates half those of the control group—even 18 months after the intervention ended. These findings collectively suggest that policymakers should consider not only the physical but also the psychological impacts of social pension programs, implementing complementary measures to help elderly recipients maintain a sense of purpose and autonomy.

Thirdly, the results in Table 3 demonstrate that the positive effect of pension income on physical health is contingent upon the local medical environment: it is evident among elderly residents in communities with better medical infrastructure but disappears where medical resources are poorer. While pension income enhances the financial capacity to seek healthcare, the actual accessibility of medical services depends critically on the availability of convenient and adequate external facilities. This finding aligns with a broader pattern observed during the COVID-19 pandemic in 2020: despite substantial fiscal allocations, many infected individuals were unable to receive timely treatment due to shortages in medical supplies. Similarly, Cheung et al. [50] documented that the effect of air pollution on mortality declined significantly and became statistically insignificant after the 2003 SARS epidemic, likely due to subsequent improvements in medical systems and community-based health services. These examples underscore that financial support alone is insufficient without corresponding investments in medical infrastructure. This study therefore highlights the need for coordinated efforts between governments and medical institutions to jointly improve the physical health of the rural elderly.

Finally, the results in Table 4 show that the negative effect of pension income on mental health is not affected by the external medical environment. Unlike explicit physical health, which is usually easy to be detected, and external medical treatment could play timely and effective role, mental health is implicit, and often invisible to older people. In addition, there is a general lack of attention to mental health among the elderly in rural China, and they rarely improve their mental health through external medical treatment. This leads to the fact that the impact of income on mental health is not affected by the external medical environment.

## 6. Conclusions and Limitations

Overall, for elderly’s physical health, money must be matched with external medical facilities to play a positive role. For mental health of the elderly, pension income plays a negative effect. Therefore, the formulation of pension policy should consider not only the positive effects on the elderly, but also the negative effects on the elderly, and realize that only money is not omnipotent in both physical and mental health of the elderly. Our study makes several key contributions to the literature. First, it enriches the body of knowledge on the income–health relationship. Unlike previous studies, we document the heterogeneous effects of pension income on the physical and mental health of the elderly. Furthermore, we identify a novel mechanism—specifically, the negative psychological cues—through which income influences mental well-being. Second, our research provides more robust empirical evidence for the income–health link. By leveraging the exogenous variation generated by China’s pension policy and constructing a rigorous Regression Discontinuity Design, we effectively address endogeneity concerns, thereby offering a more credible causal interpretation. Third, this study expands the boundaries of the income–health gradient. We demonstrate that the local healthcare environment acts as a significant moderator, indicating that adequate external medical conditions are a prerequisite for intrinsic income levels to fully exert their positive influence on health.

Our findings offer the following policy implications: (1) Reframe the pension narrative from “welfare” to “empowerment”. Public messaging should position pensions as earned rights and societal recognition of lifelong contributions, not charity. Coupled with active aging advocacy, this redefines retirement as a new life stage for growth. (2) Promote “active aging” initiatives that create meaningful social roles. Governments should support communities in developing low intensity, or volunteer activities, such as community gardening advisors or cultural heritage mentors. (3) Strengthen primary healthcare systems to effectively convert economic resources into health outcomes. This requires integrating pension policy with capacity-building for local health services.

Our study also yields valuable practical insights. For families and communities: (1) Families should reframe financial support conversations, emphasizing that pensions are “well-deserved” and enable greater autonomy, rather than reinforcing a dependent status. (2) Communities should actively foster an environment where elders “age with purpose”, recognizing their contributions to local governance, positioning them as both service recipients and community builders. For older adults themselves: (1) Redefine “labor” and “value” by translating past professional skills (farmers or craftsman) into mentorship or community teaching, shifting from “productive labor” to “legacy-building and social engagement”. (2) Proactively design post-retirement life by viewing pensions as seed funding for fulfilling lifelong dreams—such as travel or learning new skills—thereby transitioning psychologically from “working to survive” to “living for experience”.

This study acknowledges several limitations that warrant further investigation in future research: (1) Validation of psychological mechanisms. One core mechanism—the “negative psychological cues”—involves internal and sensitive mental states that are difficult to measure accurately and without bias using standard questionnaires. Future studies should adopt mixed methods approaches combining qualitative interviews and quantitative analysis. In-depth interviews and focus groups could capture the nuanced emotional and cognitive responses of the elderly toward pensions, providing richer evidence for such mechanisms and supporting the development of more effective quantitative measurement tools. (2) Cross-cultural comparative studies. Our findings are situated within a specific sociocultural context. Replicating this research in countries or regions with different cultural values (e.g., work ethics, perceptions of independence) and pension systems (e.g., universal vs. contribution-based) would help distinguish which effects are universal—driven by income itself—and which are specific to the Chinese context, thereby clarifying the boundary conditions of the proposed mechanisms. (3) Limitations in health indicators. While the study distinguishes between physical and mental health, both are multidimensional constructs. The specific metrics employed—such as certain functional scales or depression inventories—may not fully capture the comprehensive health impacts of income changes, such as improvements in chronic pain or subtle shifts in life satisfaction. Future research should incorporate broader and more objective health measures, including biomarkers (e.g., cortisol levels), medical records, and data from wearable devices on daily activity. Longitudinal designs are also needed to trace the dynamic effects of pension income on health over time, such as whether psychological impacts attenuate or persist. (4) Omitted variables and sample selection. Although the regression discontinuity design addresses key endogeneity concerns, unobserved confounders—such as concurrent local health policies that coincide with pension disbursements—may still influence the results. Additionally, sample restrictions may introduce selection bias. Future studies should seek more exogenous shocks and employ rigorous designs to further validate the income–health relationship.

## Figures and Tables

**Table 1:** Definitions of the variables and the descriptive statistics.

Variables	Definition	Whether Receiving NRPS Pension					
		Not Receiving NRPS Pension			Receiving NRPS Pension		
		N (%)	Mean	SD	N (%)	Mean	SD
PHealth	Individual’s self-assessed health status, 1 = very poor, 2 = poor, 3 = fair, 4 = good, 5 = very good.	2745 (56.6)	3.18	1.00	2101 (43.4)	3.03	0.96
1		117 (4.3)	1	0	101 (4.8)	1	0
2		411 (15.0)	2	0	414 (19.7)	2	0
3		1461 (53.2)	3	0	1131 (53.8)	3	0
4		361 (13.2)	4	0	230 (10.9)	4	0
5		395 (14.3)	5	0	225 (10.7)	5	0
MHealth	CES-D10, the score is ranging from 10 to 40. The higher the score, the better the mental health.	2745 (56.6)	32.17	6.41	2101 (43.4)	30.81	6.89
10–20		180 (6.5)	16.84	2.94	222 (10.6)	16.86	2.61
21–30		721 (26.3)	26.45	2.79	611 (29.1)	26.23	2.89
31–40		1844 (67.2)	35.90	2.76	1268 (60.3)	35.45	2.75
MEnvironment	Total number of all kinds of hospitals in community.	2745 (56.6)	2.04	2.18	2101 (43.4)	1.97	2.55
1–5		2549 (92.8)	1.60	1.25	1970 (93.8)	1.49	1.22
6–10		158 (5.8)	6.99	1.11	98 (4.6)	7.17	1.28
≥11		38 (1.4)	12.63	1.91	33 (1.6)	14.82	7.82
Age	Running variable, the age of respondents in 2015.	2745 (56.6)	52.28	6.23	2101 (43.4)	67.53	6.43
Gender	1 = male; 0 = female	2745 (56.6)	0.50	0.51	2101 (43.4)	0.53	0.53
Martial	1 = Married; 0 = Single	2745 (56.6)	0.94	0.23	2101 (43.4)	0.80	0.40
Child	LOG (the number of surviving children of the respondents)	2745 (56.6)	1.14	0.34	2101 (43.4)	1.33	0.37
Income	LOG (total income (CNY) of individual in 2014)	2745 (56.6)	9.42	1.21	2101 (43.4)	8.44	1.49
Transfer	The net amount (CNY) of (transfer payments received from children–transfer payments to children) in 2014. We take the natural logarithm of individual transfer.	2745 (56.6)	−0.35	5.93	2101 (43.4)	2.47	5.14
Twater	LOG (the quantity of tap water in local community)	2745 (56.6)	2.90	3.00	2101 (43.4)	2.95	3.00
Economic	The economic level of local community, the score ranges from 1 to 7. The higher the score, the better the economic status.	2745 (100)	3.61	1.28	2101 (43.4)	3.57	1.29

**Table 2:** RD regression results of NRPS on physical and mental health.

Panel A: The effect of NRPS on physical health				
Variables	Age Range			
	+/− 2.5	+/− 2.0	+/− 3.0	+/− 4.0
The first stage of RD				
Age	0.177 *** (3.381)	0.120 ** (2.097)	0.223 *** (4.604)	0.307 *** (7.243)
The second stage of RD				
NRPS	1.500 * (1.930)	2.687 * (1.695)	0.958 * (1.799)	0.396 (1.183)
Control variables	Yes	Yes	Yes	Yes
Obs.	963	788	1132	1421
Panel B: The effect of NRPS on mental health				
Variables	Age Range			
	+/− 2.8	+/− 2.0	+/− 3.0	+/− 4.0
The first stage of RD				
Age	0.290 *** (5.093)	0.188 *** (2.795)	0.318 *** (5.865)	0.407 *** (8.871)
The second stage of RD				
NRPS	−7.291 ** (−2.263)	−10.245 (−1.614)	−6.437 ** (−2.342)	−4.862 *** (−2.684)
Control variables	Yes	Yes	Yes	Yes
Obs.	1049	759	1110	1399

As a result of variable change, the optimal bandwidths, calculated according to Imbens and Kalyanraman [38], of physical health and mental health changes (2.5 and 2.8, respectively). The first column reports the regression results for the optimal bandwidth. In addition, we select the left and right bandwidths of the optimal bandwidths for robustness test in column (2) to column (4). All regressions controlled for Gender, Martial, Child, Income, Transfer, Tap water, Economics *** *p* < 0.01, ** *p* < 0.05, * *p* < 0.1.

**Table 3:** The impact of medical environment on the relationship between pension income and physical health.

Panel A: Communities with better medical environment				
Variables	Age Range			
	+/− 3.5	+/− 2.0	+/− 3.0	+/− 4.0
The first stage of RD				
Age	0.306 *** (4.843)	0.216 *** (2.779)	0.273 *** (4.075)	0.340 *** (5.697)
The second stage of RD				
NRPS	1.000 ** (2.047)	2.456 ** (2.160)	1.341 ** (2.197)	0.757 * (1.863)
Control variables	Yes	Yes	Yes	Yes
Obs.	591	378	532	756
Panel B: Communities with worse medical environment				
Variables	Age Range			
	+/− 3.0	+/− 2.0		+/− 4.0
The first stage of RD				
Age	0.179 *** (2.594)	0.033 (0.398)		0.279 *** (4.707)
The second stage of RD				
NRPS	0.404 (0.431)	3.969 (0.369)		−0.031 (−0.056)
Control variables	Yes	Yes		Yes
Obs.	600	410		764

As a result of sample change, the optimal bandwidths, calculated according to Imbens and Kalyanraman [38], of physical health and mental health changes (3.5 and 3.0, respectively). The first column reports the regression results for the optimal bandwidth. In addition, we select the left and right bandwidths of the optimal bandwidths for the robustness test in column (2) to column (4). Since the optimal bandwidth is exactly 3 in Panel B, there are only three columns of regression results. All regressions controlled for Gender, Martial, Child, Income, Transfer, Tap water, Economics. *** *p* < 0.01, ** *p* < 0.05, * *p* < 0.1.

**Table 4:** The impact of medical environment on the relationship between pension income and mental health.

Panel A: Communities with better medical environment				
Variables	Age Range			
	+/− 4.4	+/− 2.0	+/− 3.0	+/− 4.0
The first stage of RD				
Age	0.413 *** (6.644)	0.213 ** (2.310)	0.304 *** (3.990)	0.384 *** (5.834)
The second stage of RD				
NRPS	−4.400 * (−1.825)	−9.955 (−1.285)	−7.007 * (−1.692)	−4.976 * (−1.810)
Control variables	Yes	Yes	Yes	Yes
Obs	701	365	531	648
Panel B: Communities with worse medical environment				
Variables	Age Range			
	+/− 3.5	+/− 2.0	+/− 3.0	+/− 4.0
The first stage of RD				
Age	0.397 *** (5.794)	0.172 * (1.743)	0.339 *** (4.419)	0.433 *** (6.813)
The second stage of RD				
NRPS	−4.674 * (−1.680)	−10.32 (−1.015)	−5.578 (−1.544)	−4.530 * (−1.874)
Control variables	Yes	Yes	Yes	Yes
Obs	695	394	579	751

As a result of sample change, the optimal bandwidths, calculated according to Imbens and Kalyanraman [38], of physical health and mental health changes (4.4 and 3.5, respectively). The first column reports the regression results for the optimal bandwidth. In addition, we select the left and right bandwidths of the optimal bandwidths for robustness test in column (2) to column (4). All regressions controlled for Gender, Martial, Child, Income, Transfer, Tap water, Economics. *** *p* < 0.01, ** *p* < 0.05, * *p* < 0.1.

**Table 5:** Continuous test of control variable.

Variables	Coef.	Std. Err.	*p*
Gender	0.524	0.743	0.480
Marital	0.637	0.523	0.223
Child	0.134	0.410	0.744
Income	2.342	4.671	0.616
Transfer	−4.902	7.106	0.490
Twater	−4.237	4.591	0.356
Economic	−1.113	1.824	0.542

**Table 6:** Fake new rural insurance policy and the health of the elderly.

Panel A: Physical health				
Variables	Age range			
	+/− 8.5	+/− 7.0	+/− 5.0	+/− 3.0
NRPS	−0.066 (−0.759)	−0.061 (−0.639)	−0.024 (−0.217)	0.025 (0.177)
Control variables	Yes	Yes	Yes	Yes
Obs.	1756	1522	1153	719
Panel B: Mental health				
Variables	Age range			
	+/− 6.8	+/− 7.0	+/− 5.0	+/− 3.0
NRPS	0.171 (0.236)	0.164 (0.230)	0.116 (0.190)	0.602 (0.556)
Control variables	Yes	Yes	Yes	Yes
Obs.	1507	1522	1153	719

In this part, we assume that the age of 60 will receive a pension, but it does not. We therefore use Sharp Regression Discontinuity. The optimal bandwidth estimated by Imbens & Kalyanaraman [38] will be larger because there is no obvious jump for physical health and mental health around the age of 60.5. The first column reports the regression results for the optimal bandwidth. In addition, we select the left and right bandwidths of the optimal bandwidths for robustness test in column (2) to column (4). All regressions controlled for Gender, Martial, Child, Income, Transfer, Tap water, Economics.

## Data Availability

Data that were collected for this study can be obtained upon reasonable request by contacting the corresponding author.
